# Tissue MicroArray (TMA) analysis of normal and persistent *Chlamydophila pneumoniae *infection

**DOI:** 10.1186/1471-2334-6-152

**Published:** 2006-10-19

**Authors:** Nicole Borel, Sanghamitra Mukhopadhyay, Carmen Kaiser, Erin D Sullivan, Richard D Miller, Peter Timms, James T Summersgill, Julio A Ramirez, Andreas Pospischil

**Affiliations:** 1 Institute of Veterinary Pathology, Vetsuisse Faculty, University of Zurich, Zurich, Switzerland; 2 Division of Infectious Diseases, Department of Medicine, University of Louisville, Louisville, Kentucky, USA; 3 Department of Microbiology and Immunology, University of Louisville, Louisville, Kentucky, USA; 4 Infectious Diseases Program, School of Life Sciences, Queensland University of Technology, Brisbane, Australia; 5 Biological Defense Research Directorate, Naval Medical Research Center, 12300 Washington Avenue, Rockville, MD 20852, USA

## Abstract

**Background:**

*Chlamydophila pneumoniae *infection has been implicated as a potential risk factor for atherosclerosis, however the mechanism leading to persistent infection and its role in the disease process remains to be elucidated.

**Methods:**

We validated the use of tissue microarray (TMA) technology, in combination with immunohistochemistry (IHC), to test antibodies (GroEL, GroES, GspD, Ndk and Pyk) raised against differentially expressed proteins under an interferon-gamma (IFN-γ) induced model of chlamydial persistence.

**Results:**

In the cell pellet array, we were able to identify differences in protein expression patterns between untreated and IFN-γ treated samples. Typical, large chlamydial inclusions could be observed in the untreated samples with all antibodies, whereas the number of inclusions were decreased and were smaller and atypical in shape in the IFN-γ treated samples. The staining results obtained with the TMA method were generally similar to the changes observed between normal and IFN-γ persistence using proteomic analysis. Subsequently, it was shown in a second TMA including archival atheromatous heart tissues from 12 patients undergoing heart transplantation, that GroEL, GroES, GspD and Pyk were expressed in atheromatous heart tissue specimens as well, and were detectable morphologically within lesions by IHC.

**Conclusion:**

TMA technology proved useful in documenting functional proteomics data with the morphologic distribution of GroEL, GroES, GspD, Ndk and Pyk within formalin-fixed, paraffin-embedded cell pellets and tissues from patients with severe coronary atherosclerosis. The antibodies GroEL and GroES, which were upregulated under persistence in proteomic analysis, displayed positive reaction in atheromatous heart tissue from 10 out of 12 patients. These may be useful markers for the detection of persistent infection *in vitro *and *in vivo*.

## Background

*Chlamydophila (C.) pneumoniae *is an obligate intracellular pathogen which causes both acute and chronic respiratory infections in humans [1-5]. Over the last decade, several reports in the literature have suggested that infection with *C. pneumoniae *may also contribute to the pathogenesis of atherosclerosis [6,7]. *C. pneumoniae *was detected in atheromatous lesions by isolation in pure culture, polymerase chain reaction (PCR), electron microscopy, in situ hybridization (ISH) and immunohistochemistry (IHC) [8-11]. In order to play a causative role in chronic diseases, *C. pneumoniae *would need to persist within infected tissue for extended periods of time, thereby stimulating a chronic inflammatory response.

*In vitro*, an alteration of the normal developmental cycle of *C. pneumoniae *can be induced by interferon-γ-mediated induction of the host cell indoleamine 2,3-dioxygenase (IDO) activity, leading to a persistent form of the organism [12-15]. In addition, several other models of *in vitro *persistence have been described (i.e. iron-limitation and antibiotics) [16]. Nevertheless, it is unknown which genes and proteins of *C. pneumoniae *are involved in the development and maintenance of persistence. We have previously characterized an IFN-γ induced model of persistence by 2D gel electrophoresis [17-19], and identified several proteins that are differentially regulated during the induction of persistence.

Tissue microarray (TMA) technology, developed by Kononen et al., 1998 [20] represents a promising approach in the field of proteomics for its potential usefulness in *in situ *analysis. Preparations for TMA are constructed by obtaining cylindrical tissue specimens from paraffin blocks, assembling several hundreds into a single block, and preparing sections containing multiple tissue specimens [20-22]. TMA sections can be analyzed using standard pathology methods, such as hematoxylin and eosin (HE) staining or special stains and *in situ *analyses, such as immunohistochemistry (IHC) [20,21,23-25]. The utility of TMA protocols for high-throughput expression profiling of tumors at the molecular and protein levels has been widely used in human cancer research on formalin-fixed and paraffin-embedded biopsy specimens [20,21,26,27].

Since many markers of gene and protein expression are first established and studied in cell culture systems, it is useful to include cultured cells in TMAs for preliminary studies when translating experimental techniques from laboratory systems to studies of human tissue. Therefore, in the present study, we used 5 polyclonal antibodies directed against differentially regulated chlamydial proteins during *in vitro *persistence [18,19] to validate the use of TMA technology on non-persistently, persistently infected and uninfected HEp-2 cell pellets. In addition, archival atheromatous heart tissues [10] were tested by TMA, in combination with IHC, using the same antibodies, to determine their potential future use in detecting persistently infected tissue.

## Methods

### Cell line

HEp-2 cells (ATCC CCL-23) were obtained from the American Type Culture Collection (Rockville, MD) and maintained in Iscove's Maintenance Medium (IMM) (Cellgro, Washington, DC), as described previously [28].

### Bacterial isolate

*C. pneumoniae *A-03 (ATCC VR-1452), previously isolated from an atheroma of a patient with coronary artery disease during heart transplantation at the Jewish Hospital Heart and Lung Institute, Louisville, KY [10], were propagated in HEp-2 cell monolayers in Iscove's Growth Medium (IGM), as described previously [28]. Elementary bodies (EBs) were harvested and purified by disruption of HEp-2 cell monolayers with a cell scraper, sonication and centrifugation over a renografin density gradient [28]. EB suspensions were stored in sucrose-phosphate-glutamic acid buffer at -80°C, after which viable titers were established using standard methods.

### Patients specimens

Archival atheromatous tissue specimens from twelve patients undergoing heart transplantation were investigated. Results from culture, PCR, IHC, ISH, EM and serology testing have been described previously [10]. The study of Ramirez et al. was approved by the Institutional Review Boards (IRB) at both the University of Louisville and Jewish Hospital Healthcare Corporation.

### Preparation of antibodies

Five proteins were identified as being differentially regulated in the IFN-γ-induced model of persistence [17,19]: (i) GroEL (60 kDa chaperonin) and (ii) GroES (10 kDa chaperonin) are both chaperons involved in protein folding, assembly and modification, (iii) GspD (general secretion protein D) involved in general protein secretion, (iv) Ndk (nucleoside-2-phosphate-kinase) involved in base and nucleotide metabolism of amino acid biosynthesis, and (v) Pyk (pyruvate kinase) involved in the energy metabolism (glycolysis and gluconeogenesis). GroEL was analysed and quantitated previously [17], and the remaining four proteins were selected and confirmed in an identical fashion [19]. GroEL and GroES were upregulated under persistence, whereas GspD and Pyk remained unchanged and Ndk was downregulated. Each protein was plugged from a silver-stained 2D gel, which was obtained from a purified EB preparation [18,19,28], and used for antibody production of polyclonal antibodies at Harlan Bioproducts for Science, Inc. (Indianapolis, IN, USA). Pathogen-free, barrier-raised New Zealand white rabbits were immunized four times with the antigens, and sera from the final bleed were used in this study. The specificity of each antibody was confirmed in our laboratory by 2D gel electrophoresis of a purified *C. pneumoniae *EB preparation followed by western blot analysis, demonstrating specific reactivity on the blot which corresponded to the molecular weight and iso-electric point of each individual protein (data not shown).

### Other antibodies used

The following antibodies were also used in these studies.

(i) *Chlamydiaceae *family-specific mouse monoclonal antibody directed against the chlamydial lipopolysaccharide (mLPS; Clone ACI-P, Progen, Heidelberg, Germany).

(ii) *Chlamydiaceae *family-specific rabbit polyclonal antibody directed against both the chlamydial LPS and the chlamydial major outer membrane protein (MOMP) (pLPS/MOMP; Cygnus Technologies, Inc., Southport, NC).

### TMA analysis

#### Infection protocol

HEp-2 cells were grown in 75-cm^2 ^cell culture flasks (Costar, Cambridge, MA) to confluency and inoculated with purified *C. pneumoniae *EB (1 × 10^9 ^IFUs/flask) in IGM with or without human recombinant IFN-γ (50 and 100 U per ml), followed by centrifugation at 675 × *g *(Sorvall TR 6000D) for 30 min at 10°C and incubating at 37°C in 5% CO_2 _for 24, 48 and 72 hpi. After the respective incubation period, the medium was aspirated and the monolayers were washed twice with 1 × phosphate buffer saline (PBS). Monolayers were fixed with 4% formalin in 1 × PBS for 10 min followed by two washes with 1 × PBS. All monolayers were harvested from the flasks with a cell scraper and transferred into 15 ml of 1 × PBS. After centrifugation at 675 × *g *for 10 min at 20°C, the supernatant was discarded. The pellets were re-suspended in 5% BSA prepared in 1 × PBS and transferred to an Eppendorf™ tube (Eppendorf-Netheler-Hinz GmbH, Hamburg, Germany) with one drop of hematoxylin for visualization. The cell suspensions were centrifuged for 5 min at 950 × *g *and the supernatant was discarded. The resulting pellets were then embedded in paraffin using the Cytoblock™ cell block preparation system (Shandon™, Pittsburg, USA). For each condition (timepoints 24, 48 and 72 h, concentrations of 50 U/ml and 100 U/ml of IFN-γ) four sets of infected monolayers were prepared. As controls, four sets of uninfected HEp-2 monolayers without and with 100 U/ml IFN-γ, were prepared as pellets.

#### TMA setup

Two equal cell culture array blocks including four equal prepared sets of cell pellets were created with the TMA Builder from Histopathology Ltd., Hungary according to the instructions of the manufacturer. Briefly, the recipient paraffin block with 24 holes of diameter 2 mm each arranged in four columns and six rows was moulded with the TMA Builder. The whole cell pellets from the donor blocks were punched out with the Paraffin-Punch-Extractor and were arrayed in the preformed recipient paraffin block according to protocol.

Formalin-fixed and paraffin-embedded coronary artery specimens were available from the 12 patients (1 to 7 paraffin blocks per patient). Three TMA blocks including each tissue of the 12 patients, an uninfected and an infected control HEp-2 cell pellet (72 hpi, without IFN-γ) were created in an identical fashion. 4 μm slide were cut using a standard microtome.

#### Immunohistochemistry

Paraffin sections were stained with the following primary anti-chlamydial-antibodies: (1) mLPS at a dilution of 1:50, (2) pLPS/MOMP at a dilution of 1:400, (3) GroEL, GroES, GspD, Ndk and Pyk at a dilution of 1:200. These optimal dilutions were previously determined. Detection was performed with the Detection Kit (Dako ChemMate™ Detection Kit, Glostrup, Denmark) according to the manufacturer's instructions. Antigen retrieval was performed by one min enzyme digestion (Pronase) (mLPS and pLPS/MOMP) and microwave heating (750 W for 10 minutes) two times in citrate buffer (pH 6,0; Target Retrieval Solution (× 10), Dako ChemMate™, Glostrup, Denmark) (GroEL, GroES, GspD, Ndk and Pyk), respectively. For inhibition of the endogenous peroxidase activity, the slides were immersed in peroxidase-blocking solution (Dako ChemMate™, Glostrup, Denmark) for 5 min at room temperature (RT). Two additional blocking solutions were added to the slides, which were incubated with the polyclonal antibodies GroEL, GroES, GspD, Ndk and Pyk: Dako Protein Block Serum-free for 5 min at room temperature (Dako ChemMate™, Glostrup, Denmark) and 20 min Avidin D solution followed by 20 min Biotin solution at room temperature (Vector). The slides were incubated with the primary antibody for 60 min (mLPS and pLPS/MOMP) or over night (GroEL, GroES, GspD, Ndk and Pyk) at room temperature in a moist chamber. In total, the IHC for each antibody was repeated at least four times on consecutive sections.

## Results

### Cell pellet array

**Figure 1:**
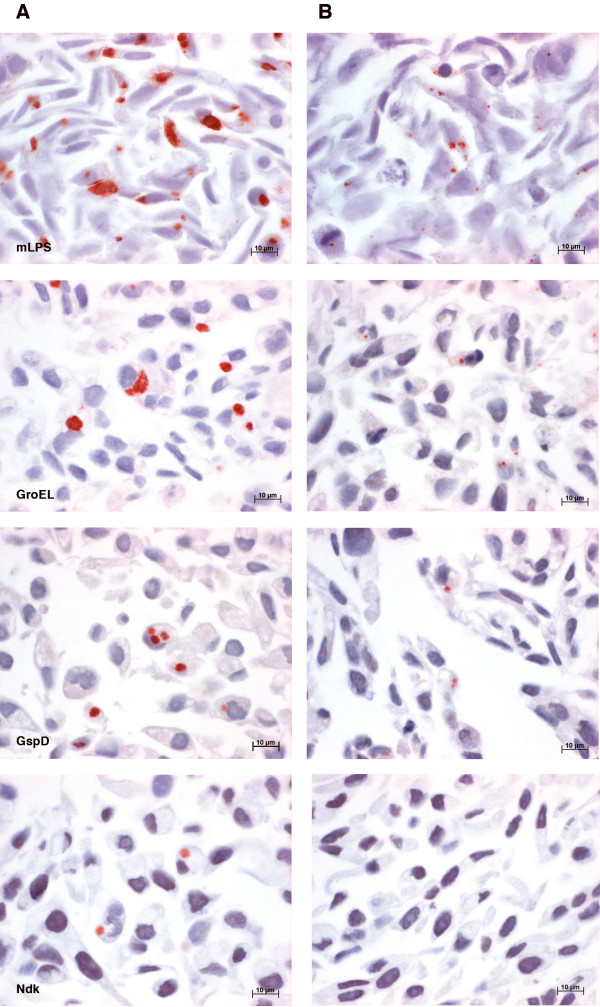
**cell pellet array**. Photomicrographs of TMA preparations of whole cell pellets showing differential expression pattern of *C. pneumoniae *proteins under untreated (A) and 50 U/ml IFN-γ treated (B) conditions at 48 hpi. Representative monolayers are shown to depict distinct differences in morphology and size of inclusions, as they were smaller and atypical under IFN-γ-induced persistence in comparison to the untreated monolayers.

Results with the monoclonal antibody directed against LPS (mLPS) and the polyclonal antibody directed against chlamydial LPS and MOMP (pLPS/MOMP) were similar. Typical large, uniformly-staining chlamydial inclusions, located near the host cell nucleus, could be seen at 48 and 72 hpi in the untreated samples with the mLPS (Figure 1) and the pLPS/MOMP antibody. Results with the 5 polyclonal antisera tested, showed similar staining patterns in the untreated samples, however, not all inclusions were stained uniformly positive (Figure 1). Earlier in the chlamydial developmental cycle at 24 h, granular positive material was seen in the cytoplasm of untreated cell pellets with the mLPS antibody and the pLPS/MOMP antibody, as well as the GroEL, GroES and Pyk antibodies, and to a lesser extent, with the GspD (data not shown). All pellets at 24 hpi were negative with the Ndk antibody.

The IFN-γ-treated samples displayed an overall decrease in positive reactivity. At 48 and 72 hpi, the number of inclusions were decreased and were obviously smaller and atypical in morphology (Figure 1). This could be observed for all antibodies, except for Ndk, which was negative in all IFN-γ-treated samples (Figure 1). There was no difference in inclusion morphology and antibody staining patterns seen between the two concentrations of IFN-γ (50 U/ml or 100 U/ml). Overall, the assay showed good reproducibility in all four replicates of monolayers.

### TMA with atheromatous heart tissues

Granular, positive-staining material was seen in the cytoplasm of subintimal macrophages and smooth muscle cells in the medial part of the affected coronary arteries of all patients with at least one antibody, except the two patients with negative results for the presence of *C. pneumoniae *in the original study (patients # 7 and 11) [10]. Patients # 10 and 12 were positive with all antibodies used except of the Ndk, which was negative in all 12 heart tissue specimens. Patients # 1, 2, 3 and 9 were positive with the GroEL, GroES, GspD and Pyk, whereas patients # 4 and 6 were only positive with the GroEL, GspD and Pyk. Patient # 5 was positive with the GroES and Pyk, and patient # 8 revealed positive reaction with the GroEL and GspD. All patients were negative when tested with the mLPS, whereas the pLPS/MOMP ab revealed positive reactions in patients # 10 and 12.

**Figure 2:**
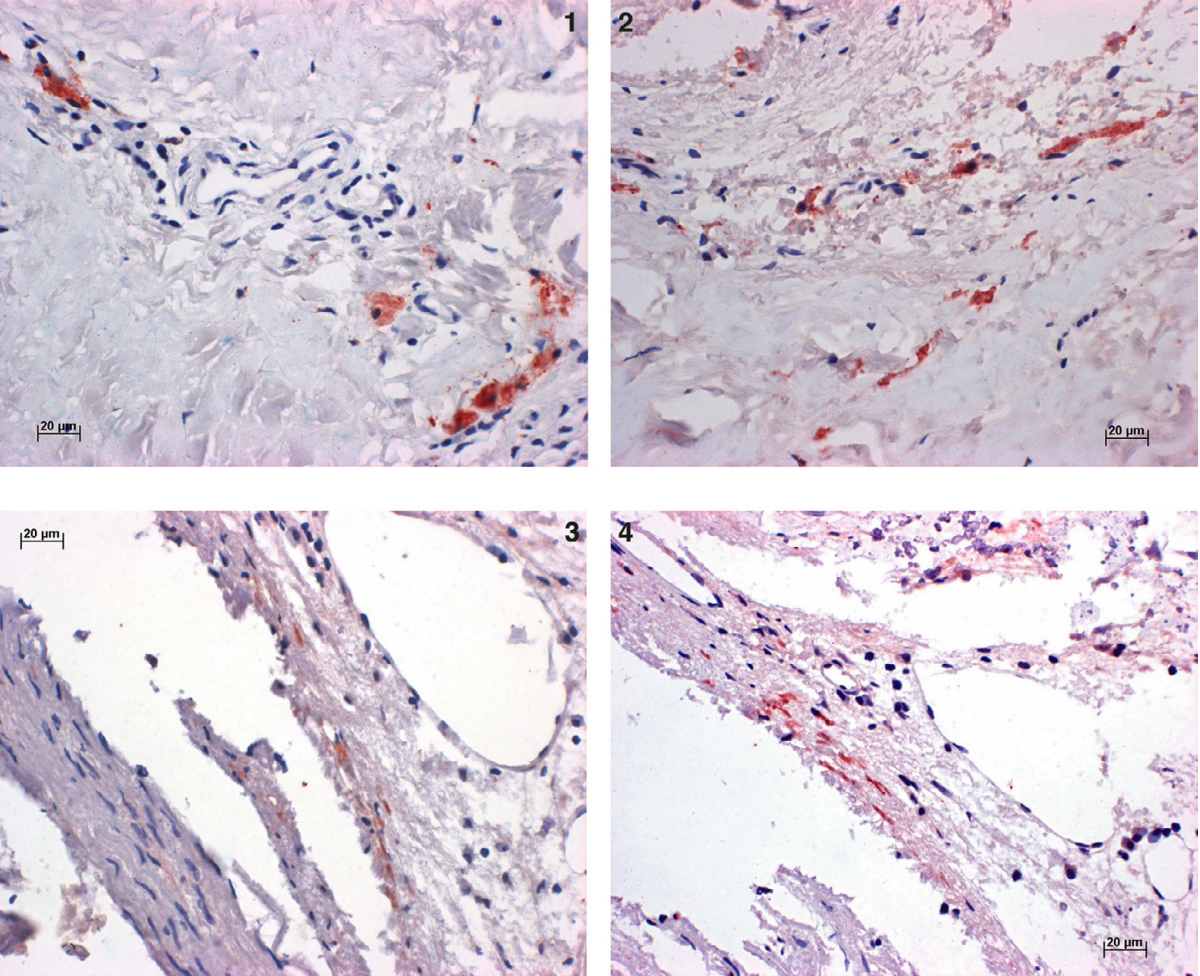
**TMA with atheromatous heart tissues**. Photomicrographs of TMA preparations of atheromatous heart tissues of patient # 10 showing positive reaction within macrophages with GroEL (1) and with GroES (2) and in patient # 12 showing positive reaction within smooth muscle cells with GspD (3) and Pyk (4).

Positive staining of the antibodies GroEL and GroES in patient # 10 and GspD and Pyk in patient # 12, respectively, is displayed in figure 2.

## Discussion

In this study, TMA technology was useful in documenting functional proteomics data showing the morphologic distribution of GroEL, GroES, GspD, Ndk and Pyk within formalin-fixed, paraffin-embedded cell pellets and atheromatous heart tissues. GroEL, GroES, GspD, Ndk and Pyk, selected as differentially regulated proteins from proteomic analysis, were expressed in *C. pneumoniae-*infected, untreated and IFN-γ treated HEp-2 cells and atheromatous heart tissues and were detectable morphologically by IHC. In general, IHC allows the detection of the presence of an antigen in tissue sections, however, the intensity of antigen labelling does not correlate with the amount of antigen present. The IHC labelling evaluated in this study, therefore, represents the presence or absence of proteins, but does not reflect quantitative expression of GroEL, GroES, GspD, Ndk, Pyk, mLPS and/or pLPS/MOMP.

By using cell pellet array, in combination with IHC, it was possible to identify the number, size and morphology of the chlamydial inclusions. Numbers of inclusions at 48 hpi and 72 hpi were decreased under IFN-γ-persistence and the inclusions were smaller and atypical, as described previously [15], however, the staining intensity remained the same as that seen in untreated cells.

The staining results obtained with the TMA method were generally similar to the changes observed between normal and IFN-γ persistence using proteomic analysis [17-19]. For example, GroEL, which was upregulated in proteomic analysis, displayed more positive reactions in the IFN-γ treated samples when compared to GspD, which remained unchanged in the proteomic analysis. Likewise, Ndk reactivity remained negative in all IFN-γ-treated samples by TMA, which corresponded to the downregulation observed in the proteomic analysis.

In comparison to other methods, such as PCR, we were able to localize and visualize the positive reactions within the atheromatous heart tissue by TMA, in combination with IHC. The TMA method allowed a comparison between multiple cell pellets or tissue specimens on one microscope slide. The 2 mm punch, in combination with the Tissue Microarray Builder allowed easy manipulation during punching, facilitating a rapid preparation of the cell culture array block. The fact that the 2 mm punch can cause significant damage to the donor block was not of importance because each donor block contained only one single cell pellet that was entirely punched out and the whole area of each pellet could be examined. Other punch sizes such as 0.6 mm and 1.2 mm cause less damage to the original donor block and make it possible to array several hundreds of specimens on one single block, however the creation of this block is more laborious. The problem of reduction of the amount of tissue analyzed from the whole cell pellet to a disk, which may be not be representative of the protein expression of the entire tissue specimen, can be solved by performing the experiments in several-fold redundancy. For that reason, we created two equal cell pellet blocks including four sets of equally prepared monolayers containing the three timepoints (treated and untreated cell pellets and negative controls). Multiple sections of the two TMA blocks were cut and probed with each antibody.

Multiple sections of 3 TMA blocks, including the atheromatous heart tissue, were investigated with each antibody. In our previous study, patients # 1, 2, 3, 6 and 12 were positive by IHC using Chlamydia-specific and *C. pneumoniae*-specific antibodies [10]. We were able to detect 5 more positive patients with experimentally produced anti-GroEL, anti-GroES, anti-GspD and anti-Pyk antibodies. Thus, differentially regulated proteins by proteomic analysis were expressed in *C. pneumoniae*-infected human atheromatous heart tissue specimens and were detectable morphologically within lesions by IHC. The staining results obtained with the TMA corresponded to the reactivity as determined by proteomic analysis, for example, GroEL and GroES, which were upregulated under persistence in proteomic analysis, were likewise positive in most heart tissue specimens. Patients # 7 and 11, which underwent heart transplantation primarily due to myocarditis, rather than severe atherosclerosis (unpublished data), displayed questionable positive results (GroEL, GroES, GspD and Pyk). As these 2 patients were in fifth decade of life and had elevated anti-*C. pneumoniae *titers in the microimmunofluorescence assay [10], we tend to assume that they suffered from atherosclerosis and were Chlamydia-infected as patients # 1–6, 10 and 12, but perhaps to a lesser extent. Definitive demonstration of chlamydial particles in patients # 7 and 11 by more sensitive techniques (i.e. ultrastructural studies) is in progress [29].

## Conclusion

Overall, GroEL and GroES tend to be useful markers to detect persistent infection *in vitro *and *in vivo*. In addition, GspD and Pyk antibodies gave similar reactivity, indicating appropriate sensitivity and specificity, and may also allow the detection of *C. pneumoniae *in chronically-infected tissue. Antibody prepared against Ndk remained negative in tissue specimens from all 12 archived atheromatous tissue specimens, which corresponded nicely to the downregulation observed in proteomic analysis. These data represent the first thorough examination of atheromatous tissue by experimentally produced antibodies against *C. pneumoniae *and may provide a useful technique to further define the role of this organism in atherosclerosis and other chronic human diseases.

## Competing interests

The author(s) declare that they have no competing interests.

## Authors' contributions

NB carried out the TMA analysis and drafted the manuscript. SM performed the cell culture infections and the 2 D gel electrophoresis. CK performed the IHC. EDS participated in the cell culture infections and 2 D gel electrophoresis. RDM and PT participated in the coordination of the study and helped to draft the manuscript. JTS and AP participated in the design of the study. JAR provided the atheromatous heart tissue specimens. All authors read and approved the final manuscript.

## Pre-publication history

The pre-publication history for this paper can be accessed here:


